# Time-Dependent Effects of Acute Exercise on University Students’ Cognitive Performance in Temperate and Cold Environments

**DOI:** 10.3389/fpsyg.2017.01192

**Published:** 2017-07-12

**Authors:** Ling-Yu Ji, Xiao-Ling Li, Yang Liu, Xiu-Wen Sun, Hui-Fen Wang, Long Chen, Liang Gao

**Affiliations:** ^1^School of Mechanical Engineering, Xi’an Jiaotong University Xi’an, China; ^2^China Research and Development Academy of Machinery Equipment Beijing, China

**Keywords:** acute exercise, processing speed, working memory, cognitive flexibility, time-dependent, temperate, cold

## Abstract

**Background:** Few studies have examined the acute exercise-induced changes in cognitive performance in different thermal environments and the time course effects.

**Objective:** Investigate the time-dependent effects of acute exercise on university students’ processing speed, working memory and cognitive flexibility in temperate and cold environments.

**Method:** Twenty male university students (age 23.5 ± 2.0 years) with moderate physical activity level participated in a repeated-measures within-subjects design. Processing speed, working memory and cognitive flexibility were assessed using CogState test battery at baseline (BASE), followed by a 45-min rest (REST), immediately after (EX) and 30 min after (POST-EX) 30-min moderate-intensity treadmill running in both temperate (TEMP; 25°C) and cold (COLD; 10°C) environments. Mean skin temperature (MST) and thermal sensation (TS) were also recorded. Two-way repeated measures ANOVA was performed to analyze each variable. Spearman’s rho was used to identify the correlations between MST, TS and cognitive performance.

**Results:** Reaction time (RT) of processing speed and working memory decreased immediately after exercise in both conditions (processing speed: *p* = 0.003; working memory: *p* = 0.007). The facilitating effects on processing speed disappeared within 30 min after exercise in TEMP (*p* = 0.163) and COLD (*p* = 0.667), while improvements on working memory remained 30 min after exercise in TEMP (*p* = 0.047), but not in COLD (*p* = 0.663). Though RT of cognitive flexibility reduced in both conditions (*p* = 0.003), no significance was found between EX and REST (*p* = 0.135). Increased MST and TS were significantly associated with reductions in processing speed RT (MST: *r* = -0.341, *p* < 0.001; TS: *r* = -0.262, *p* = 0.001) and working memory RT (MST: *r* = -0.282, *p* < 0.001; TS: *r* = -0.2229, *p* = 0.005), and improvements in working memory accuracy (MST: *r* = 0.249, *p* = 0.002; TS: *r* = 0.255, *p* = 0.001).

**Conclusion:** The results demonstrate different time-dependent effects of acute exercise on cognition in TEMP and COLD. Our study reveals facilitating effects of exercise on university students’ processing speed and working memory in both environments. However, in contrast to TEMP, effects on working memory in COLD are transient.

## Introduction

Despite the fact that regular participation in physical activity (PA) is associated with physical and mental health benefits (Vankim and Nelson, 2013; Pedišić et al., 2014), physical inactivity has become one of the major health problems of university students around the world (Pengpid et al., 2015). Besides physical and mental health, PA is also positively related to cognitive functions (Kamijo and Takeda, 2009; Stroth et al., 2009; Cirillo et al., 2017) and academic performance (Kwak et al., 2009). Thus, it is necessary for university students to promote PA level in their everyday lives.

In addition to the long-term effects of PA, a vast number of studies have also demonstrated the facilitating effects of a single bout of acute exercise on cognitive performance (Lambourne and Tomporowski, 2010; Chang et al., 2012). Evidence has been presented indicating that academic performance is associated with processing speed (Rohde and Thompson, 2007), working memory (Bergman Nutley and Söderqvist, 2017) and cognitive flexibility (Latzman et al., 2010). Thus, an acute exercise intervention between study sessions may yield positive effects on university students’ learning efficiency. However, results from studies on the effects of acute exercise on these types of cognitive functions are inconsistent. In addition, changes in the seasonal thermal environmental conditions may also influence cognitive function. Cognitive performance is impaired during and after exposure to hot and cold environmental conditions (Hancock et al., 2007; Taylor et al., 2015). According to a meta-analytical review by Hancock et al. (2007), decline in cognitive performance is larger when the air temperature is below 11.1°C than under heat stressor, indicating that cognitive performance may be worse in winter than in other seasons. Therefore, it will be much meaningful to take different thermal environmental conditions (temperate and cold) into consideration while investigating the effects of acute exercise on university students’ cognitive functions.

Processing speed refers to the efficiency with which cognitive tasks, particularly elementary cognitive tasks, are executed (Kail and Salthouse, 1994). A majority of the previous studies have used choice reaction time (CRT) paradigms to assess processing speed (Salthouse, 2000). There is clear evidence that acute exercise has a positive impact on the performance of CRT tasks (Davranche and Audiffren, 2004; Kashihara and Nakahara, 2005; Audiffren et al., 2008). Draper et al. (2010) also found a significant linear decrease in CRT when exercise intensity increases, without compromising accuracy. While there’re plenty of studies conducted in laboratory settings at room temperature, to our knowledge, only one study has investigated the effect of exercise on CRT in cold environments. Kruk et al. (2001) reported that compared to rest, ergometer cycling at an intensity of 200W shortened CRT when exposure to 4°C. However, the generalizability of this study may be questioned as they only examined elite soccer players. Since effects of acute exercise on RT are correlated with individual’s aerobic fitness (Bullock and Giesbrecht, 2014), further investigation in university students with moderate fitness level is still needed.

Working memory refers to a limited capacity system that allows holding and manipulating temporal information (Baddeley, 2000) and is one of the core components of executive functions (Diamond, 2013). Previous studies have shown positive (Pontifex et al., 2009; Quelhas Martins et al., 2013), negative (Coles and Tomporowski, 2008) or no effects (Tomporowski et al., 2005) of a short-term exercise on working memory. Results from two meta-analytical reviews (Chang et al., 2012; Roig et al., 2013) indicated that acute exercise had small to moderate effects on working memory, in general. It seems that subjects’ fitness level, mode of exercise, exercise duration and intensity and the time of cognitive test administration might be the potential moderators. Taken together, aerobic exercise at low to moderate intensity with the cognitive test administrated after exercise in adults with moderate fitness level seems to have the largest effects. While evidence has been shown that working memory is impaired in cold stress (Mahoney et al., 2007; Muller et al., 2012), no published studies have reported on the effects of exercise on working memory in cold environmental conditions.

Another core component of executive functions is cognitive flexibility, which involves changing between multiple tasks, operations or mental sets (Monsell, 1996). Cognitive flexibility is often investigated using set-shifting, or task switching tasks (Diamond, 2013). Inconsistencies have also been found from previous studies on the effects of acute exercise on cognitive flexibility. Two studies that failed to find facilitating effects used similar moderate ergometer cycling protocol and found improvements in set-shifting in both exercise and control group (Coles and Tomporowski, 2008; Wang et al., 2015), while studies implemented high intensity exercise showed positive effects following exercise, compared to control group (Barenberg et al., 2015; Berse et al., 2015), indicating that exercise intensity might be a crucial moderator. However, a recent study also found a facilitating effect in young adults with high fitness level using moderate exercise protocol (Tsai et al., 2016). Thus, the evidence of acute exercise effects on cognitive flexibility is still inconclusive. So far, no empirical study has examined cognitive flexibility performance in cold environments in humans.

The sustained effects are also of practical importance when investigating the impact of acute exercise intervention on cognitive functions. Based on the results of previous literature examining the time-dependent, or time course effects, facilitation of higher-level cognitive functions following exercise seems to last longer than of lower mental processes. Pontifex et al. (2009) reported that exercise-induced arousal on working memory could still exist 30 min after the cessation of exercise. Another study examining response execution and response inhibition reported a positive effect that sustained up to 52 min following exercise (Joyce et al., 2009). While for CRT, results from two studies showed the arousing effects disappeared immediately after exercise or within 8 min (Kashihara and Nakahara, 2005; Audiffren et al., 2008). While in cold environments, changes in cognitive performance might be more complex, since the negative impacts of cold stress may adversely affect the sustention of exercise-induced arousal. Muller et al. (2011) found a decrease in selective attention performance after a 45-min recovery in 5°C, compared to 5 min after the termination of exercise.

Previous studies conducted in cold and temperate environments also presented a correlation between cognitive performance and subject’s Mean skin temperature (MST) and thermal sensation (TS) when subjects were seated or performing small body movements (Palinkas et al., 2005; Mäkinen et al., 2006; Watkins et al., 2014). Human and animal studies have suggested that significant reduction in body temperature would cause decline in cerebral blood flow and cerebral oxygen consumption (Laptook et al., 2001; Pozos and Danzl, 2001; Li et al., 2009), which would cause cognitive deficits (Raichle et al., 2001). This could be a possible explanation of the mechanism underlying the correlations between cognition, MST and TS in cold and temperate environments. Since exercise can cause increase in body temperature and have benefits on cognitive functions, it can be hypothesized that MST and TS may also be associated cognitive performance when people perform more vigorous exercise.

The present study aimed to contribute to orientate the research on the following aspects regarding the time-dependent effects of acute exercise on university student’s cognitive performance in temperate and cold environments: (1) Although the benefits of exercise intervention on cognitive performance in temperate environment have been well-examined by previous studies, cold environment has been poorly investigated. It is also meaningful to compare cognitive performance in different thermal conditions to take appropriate measures to improve student’s learning efficiency. Thus, we investigated the effects of acute exercise on processing speed, working memory and cognitive flexibility in temperate and cold environments. (2) The time course of the facilitation effects determines how long the student can benefit from acute exercise. Given the lack of investigation on the time-dependent effects, this study examined the time course with the cognitive tests administrated before, immediately following and after a delay following exercise. (3) Because previous studies which suggested correlations between cognitive performance and MST and TS didn’t include an acute exercise intervention, we aimed to add to the literature by examining the association before, immediately after and after a delay following exercise.

## Materials and Methods

### Participants

A convenience sample of twenty (*n* = 20) young adult males (mean age 23.5 ± 2.0 years, range 21–28 years; height 174.4 ± 2.9 cm, body mass 66.7 ± 5.8 kg) who were undergraduate or graduate students at Xi’an Jiaotong University volunteered for this study and were paid for the participation. Only males were included due to gender differences in thermoregulation and exercise capacity (Kaciuba-Uscilko and Grucza, 2001). Exclusion criteria were history of psychiatric, neurological, cardiac or metabolic diseases, vigorous exercise during the last 24 h or <12 h of sleep during the last 48 h prior to the test. Participant’s fitness level was estimated by PA level using International Physical Activity Questionnaire (IPAQ) (Craig et al., 2003), which has been shown to be significantly associated with VO_2max_ (Mynarski et al., 2009; Papathanasiou et al., 2010). All participants were classified as “moderate physical activity” (600–3000 MET-minutes/week). Participants also completed American College of Sports Medicine (ACSM) risk stratification questionnaire and were classified as “low risk” according to risk stratification criteria (American College of Sports Medicine [ACSM], 2013). The study protocol was approved by the Institutional Review Board at Xi’an Jiaotong University. All participants gave their written informed consent in accordance with the Declaration of Helsinki. One participant was excluded from further analysis due to technical reasons. Therefore, all 20 participants finished the study protocol, but only 19 participants are included in the data analyses.

### Study Design and Procedures

**FIGURE 1:**
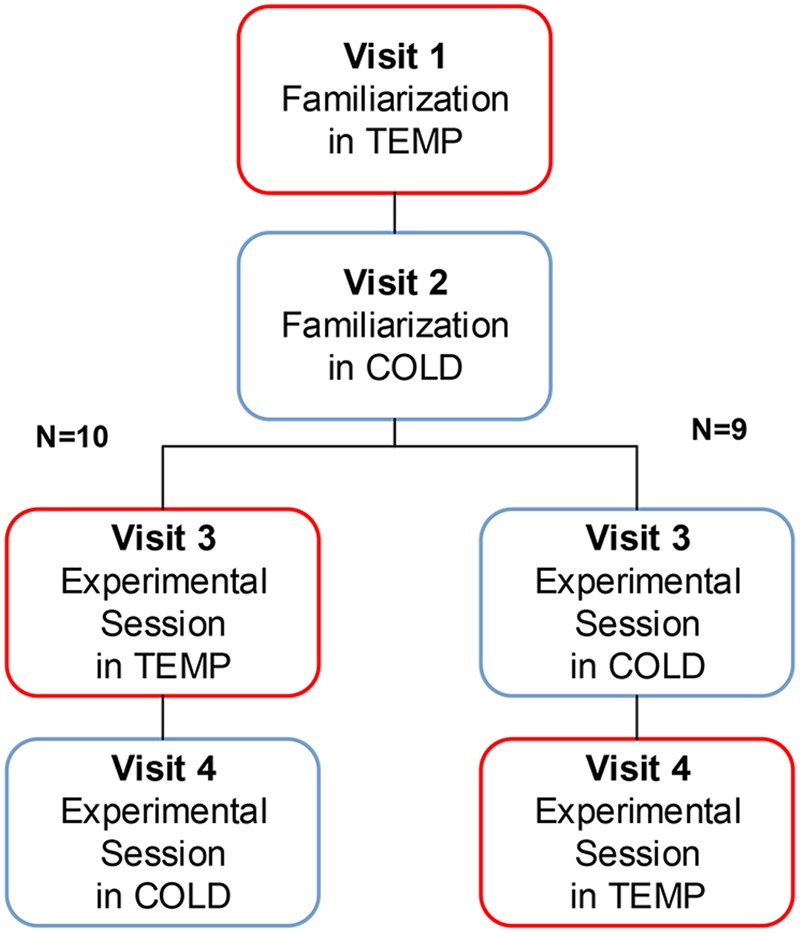
Overview study design.

This study employed a repeated-measures, within-subjects design. Participants reported to the laboratory on four separate occasions, including two familiarization visits and two experimental sessions (**Figure 1**).

The familiarization visits were to collect anthropometric data and for familiarization to the cognitive tests and different thermal conditions. On Visit 1, participant’s age and anthropometric data [height, body mass, resting heart rate (HRrest)] were collected. Participants’ maximum HR (HRmax) was also estimated using the formula ‘HRmax = 220–age’ to calculate their target HR in the exercise session (American College of Sports Medicine [ACSM], 2013). Then participants sat in an environmental chamber in a temperate thermal condition [TEMP; 25°C, 50% relative humidity (RH)] and practiced the cognitive tests three times. Experimental protocol and physiological measurement procedures were also familiarized. Participants walked at 5 km/h for 2 min on a motorized treadmill (Cybex International, Inc., Medway, MA, United States). Then participants completed the treadmill running protocol which was same as in the experimental sessions. On Visit 2, participants practiced the cognitive tests three times and familiarized with the experimental protocol in the environmental chamber in cold condition (COLD; 10°C, 50% RH). The temperature 10°C was selected according to the winter indoor temperature in southern China which was defined as “Hot summer & cold winter climate zone,” ranging from 8 to 14°C (Yoshino et al., 2006; Luo et al., 2016).

For experimental sessions, in order to minimize learning effects, counterbalanced design was employed. The order of the experimental sessions for different individuals was randomly drawn by computer. All participants underwent both TEMP and COLD. Ten participants underwent TEMP then COLD, while nine underwent COLD then TEMP. Each of the four visits was separated by 7 days.

### Experimental Sessions

**FIGURE 2:**
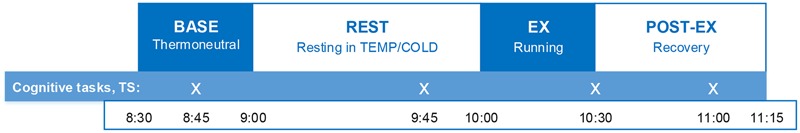
Experimental protocol.

All the experimental sessions were conducted from 08:30 to 11:15 a.m. Participants were asked to wear a long-sleeved sweater, straight trousers, long underwear bottoms, long underwear top and athletic shoes for test conditions. Each session consisted of a 30-min baseline period (BASE), 60 min of rest (REST), 30 min of exercise (EX) and a 45-min post-exercise period (POST-EX) (**Figure 2**).

Baseline data (TS, cognitive tests) were collected in the laboratory where room temperature was approximately 20°C. The first set of cognitive tests were administrated 15 min prior to the ending of BASE. The duration of each set of cognitive tests was 13–15 min. Once completed, participants entered the environmental chamber and underwent either TEMP or COLD. Participants remained seated on an office chair, reading academic books or articles for 45 min which was scheduled according to the length of a single class in Chinese universities (40–50 min). After the cessation of REST, participants reported TS and performed the second cognitive test followed by EX.

The EX employed a moderate-intensity treadmill running protocol. The exercise intensity was determined in accordance with results from a previous meta-analytical review (McMorris and Hale, 2012) which suggested that moderate intensity exercise had larger benefits on cognition than low and high intensities. Participants performed a 3-min warm-up at 5 km/h on the treadmill. Then the speed increased (within 2–3 min) until participants reached their target HR, which was 60% of their heart rate reverse (HRR = HR_max_-HR_rest_). Participants kept running at their predetermined target HR for approximately 23 min, followed by a 2-min cool-down period. Immediately after the cessation of running, participants sat on the office chair, reported TS and performed the third set of cognitive tests. The final TS report and cognitive test were completed following another rest period, which lasted for 15 min.

### Measurements

#### Heart Rate (HR)

Heart Rate was measured with a Polar H7 HR monitor which was connected to the Polar Beat App (Polar, Polar Electro, Finland) across the entire experimental session.

#### Mean Skin Temperature (MST)

Skin temperatures were recorded using eight iButton skin thermochrons (Maxim Integrated, San Jose, CA, United States) which were placed on the forehead, chest, upper arm, forearm, hand, anterior thigh, anterior calf and foot of the right side of the body and were attached with medical tape. Skin temperature of each site was recorded every 5 min throughout the experimental session. MST was calculated using Gagge/Nishi’s equation (Nishi and Gagge, 1970):

$$T_{\text{sk}}\;=\;0.07T_{\text{forehead}}+0.175T_{\text{chest}}+0.175T_{\text{upperarm}}+0.07T_{\text{forearm}}+0.07T_{\text{hand}}+0.05T_{\text{calf}}+0.19T_{\text{thigh}}+0.2T_{\text{foot}}$$

#### Thermal Sensation (TS)

Thermal sensation involves the feelings (hot, neutral, cold) resulting from exposure to various ambient temperatures. In this study, TS was assessed using a 7-point scale (from -3 = cold to +3 = hot) (ASHRAE, 2010).

#### Cognitive Tests

The CogState computerized test battery^1^ was used to assess cognitive performance. The CogState test battery has been shown good reliability and validity with most of the tasks use playing cards stimuli. Three tasks were chosen from the battery, including Identification for processing speed, Two Back for working memory and Set-Shifting for cognitive flexibility. Outcome measures include speed, which is measured as the log10-transformed RT, and accuracy.

##### Identification (processing speed)

Identification task measures processing speed using a CRT paradigm. A playing card is presented in the center of the screen in a face-down condition. Once the card flips over, participants must decide whether it’s a red card or black card. If it’s red, participants should press the “Yes” key by clicking on the left button of the mouse. If it’s black, participants should press the “No” key by clicking on the right button. Participants should respond to the color stimuli as soon as possible. Total duration of the task is 3 min.

##### Two back (working memory)

Two Back task uses a n-back paradigm to measure working memory. A playing card is shown in the center of the screen in a face-up condition. Participants must hold information of the previous two cards and decide whether the current card is the same as the card that was shown two cards ago. If it is, then participants should press “Yes.” If it’s not, participants should press “No.” Participants have to be quick and accurate in responding. Total duration of the task is about 4 min.

##### Set-shifting (cognitive flexibility)

Set-Shifting task measures cognitive flexibility using a set shifting paradigm. In Set-Shifting task, there’s two kinds of stimuli: the color and the number of the playing card. The participants are asked, “Is this the target card?” A playing card is shown in the center of the screen in a face-up condition with the word “Color” or “Number” above it. Participants must guess whether the target card is red or black, or whether the number of the current card is the target number. In the beginning, participants just need to guess whether the first card is the target card. The next card will not be shown until participants make the correct response. Then participants must keep the target color or number in mind, decide whether the next set of cards match the target color or number and make response until the hidden rule changes (e.g., from one number to another [intra-dimensional shift], or from number to color [extra-dimensional shift]). Participants have to work as quickly and as accurately as possible. Total duration of the task is about 7 min.

### Statistical Analysis

Statistical analysis was performed using SPSS 24.0 (IBM, New York City, NY, United States). Two way (condition × time point) repeated measures ANOVA was performed to analyze each of the dependent variables: MST, TS and the log10-transformed RT and accuracy of the cognitive tests. Mauchly’s test of sphericity was performed and any data indicated a violations of sphericity were adjusted using Greenhouse-Geisser adjustment. Bonferroni’s *t*-test was also used in the further *post hoc* test to determine where significance occurs. To identify the correlations between MST, TS and cognitive performance, Spearman’s rho was utilized because the data of accuracy on cognitive tests were found to violate assumption of normality. Significance level was set at α < 0.05.

## Results

### Heart Rate

Participants’ mean HR_rest_ and HR during exercise in TEMP and COLD are shown in **Table 1**. During the exercise period, participants’ HR reached 61.96 ± 3.77% and 62.19 ± 3.95% of their HRR in TEMP and COLD, respectively, suggesting that the desired moderate exercise intensity was achieved.

**Table 1:** HR_rest_, HR (bpm) and %HRR during exercise in TEMP and COLD (Mean ± SD).

Condition	HR_rest_	HR during exercise	%HRR
TEMP	77.11 ± 5.93 0.133	150.84 ± 6.34 0.303^∗∗∗^	61.96 ± 3.77
COLD	78.95 ± 6.21 0.061	151.89 ± 6.44 0.344^∗∗∗^	62.19 ± 3.95 0.065

### Mean Skin Temperature

**FIGURE 3:**
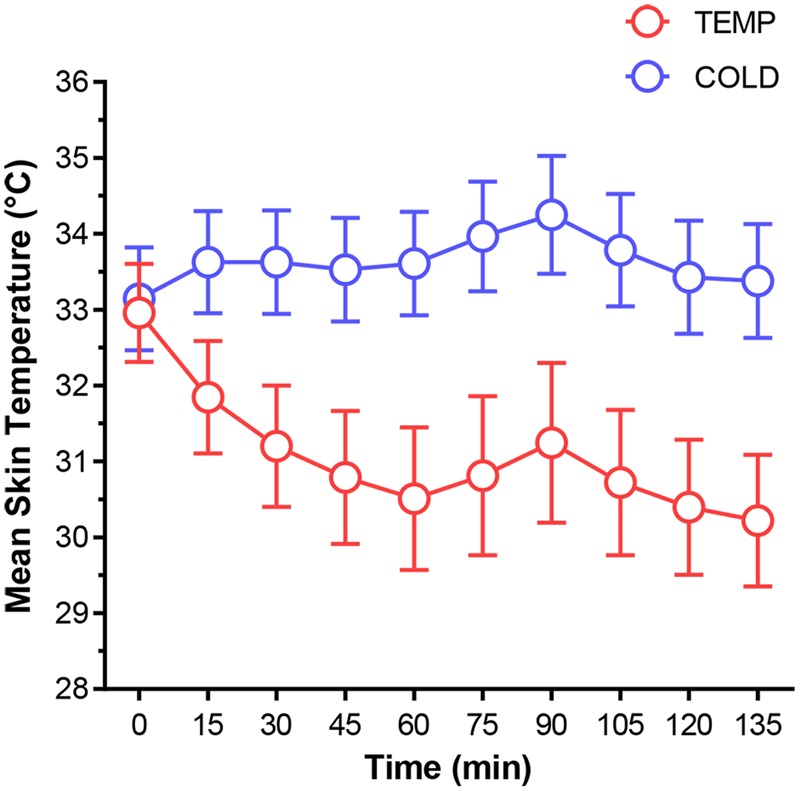
Mean skin temperature for both environmental conditions. Error bars represent SD.

Repeated measures ANOVA revealed a significant main effect for condition [*F*(1,18) = 141.85, *p* < 0.001, $\eta_{p}^{2}$ = 0.887)], where mean *T*_sk_ was 2.56°C lower in COLD than TEMP (95% *CI* = -3.02 to -2.11, *p* < 0.001) (**Figure 3**). A significant condition × time interaction effect was also observed [*F*(9,162) = 107.81, *p* < 0.001, $\eta_{p}^{2}$ = 0.857)], where *T*_sk_ in TEMP slightly rose from 33.14 ± 0.68°C to 33.61 ± 0.68°C during REST, whereas in COLD there was a 2.45°C reduction (**Figure 3**).

### Thermal Sensation (TS)

**FIGURE 4:**
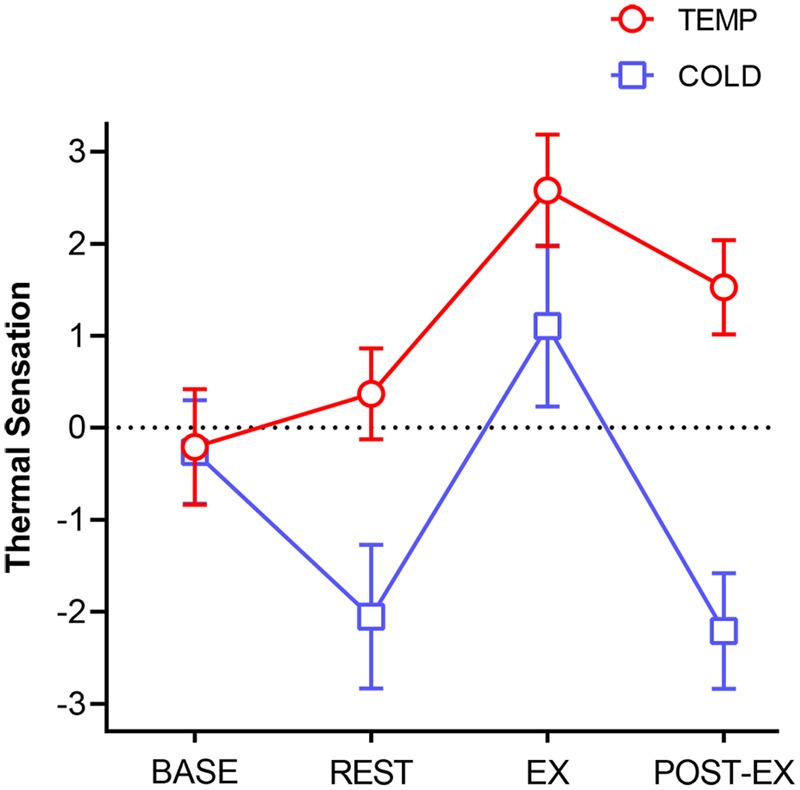
Thermal sensation for both environmental conditions. Error bars represent SD.

A significant main effect for condition [*F*(1,18) = 559.31, *p* < 0.001, $\eta_{p}^{2}$ = 0.969)] was found for TS, where mean TS was 1.92 units lower in COLD than in TEMP (95% *CI* = -2.09 to -1.75, *p* < 0.001). A significant condition × time interaction effect [*F*(3,54) = 91.77, *p* < 0.001, $\eta_{p}^{2}$ = 0.836)] showed the different change tendencies in TEMP and COLD over the 135 min (**Figure 4**).

### Cognitive Performance

**FIGURE 5:**
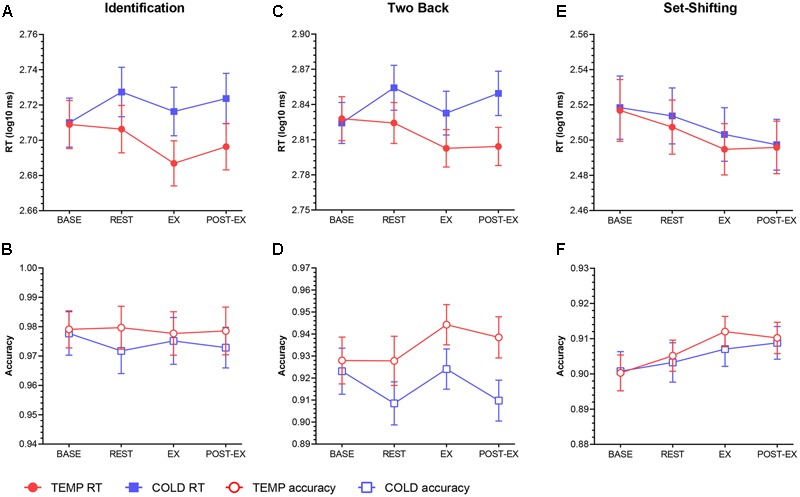
Mean RT and accuracy data for Identification **(A,B)**, Two back **(C,D)** and Set-shifting **(E,F)** tasks. Error bars represent SEM.

Results for cognitive performance are shown in **Figure 5**.

The mean RT data for the Identification task are shown in **Figure 5A**. Repeated measures ANOVA revealed a main effect on reaction time (RT) for condition [*F*(1,18) = 6.44, *p* = 0.021, $\eta_{p}^{2}$ = 0.264] and time [*F*(3,54) = 2.94, *p* = 0.041, $\eta_{p}^{2}$ = 0.140]. Pairwise comparisons demonstrated significant reductions between REST and EX in both conditions (95% *CI* = -0.026 to -0.004, *p* = 0.003). However, no significant difference was found between REST and POST-EX in either TEMP (95% *CI* = -0.024 to 0.004, *p* = 0.163) or COLD (95% *CI* = -0.021 to 0.014, *p* = 0.667). There was also a significant condition × time interaction [*F*(3,54) = 3.163, *p* = 0.032, $\eta_{p}^{2}$ = 0.149], which was driven by the significant increase in RT in COLD between BASE and REST (95% *CI* = 0.005 to 0.030, *p* = 0.011).

The accuracy data are shown in **Figure 5B**. There was no main effect for condition [*F*(1,18) = 1.11, *p* = 0.305, $\eta_{p}^{2}$ = 0.058], time [*F*(3,54) = 0.07, *p* = 0.978, $\eta_{p}^{2}$ = 0.004] or condition × time interaction [*F*(3,54) = 0.11, *p* = 0.955, $\eta_{p}^{2}$ = 0.006].

For Two Back Task, the RT data are shown in **Figure 5C**. A significant main effect for condition [*F*(1,18) = 6.70, *p* = 0.019, $\eta_{p}^{2}$ = 0.271] and a condition × time interaction [*F*(3,54) = 5.23, *p* = 0.003, $\eta_{p}^{2}$ = 0.225] were observed. *Post hoc t*-tests revealed that RT decreased between REST and EX in both conditions (95% *CI* = -0.041 to -0.002, *p* = 0.007). Further analysis found a significant decrease between REST and POST-EX in TEMP (95% *CI* = -0.020 to 0.009, *p* = 0.047), but no significant difference in COLD (95% *CI* = -0.005 to 0.011, *p* = 0.663). There was no main effect for time [*F*(3,54) = 1.88, *p* = 0.144, $\eta_{p}^{2}$ = 0.094].

Result for accuracy (**Figure 5D**) demonstrated a significant main effect for condition [*F*(1,18) = 4.78, *p* = 0.042, $\eta_{p}^{2}$ = 0.210]. There was no main effect for time [*F*(3,54) = 1.21, *p* = 0.314, $\eta_{p}^{2}$ = 0.063] or condition × time interaction [*F*(3,54) = 1.46, *p* = 0.236, $\eta_{p}^{2}$ = 0.075]. No significant difference was found between each two time points in both conditions.

For Set-Shifting Task, the results (**Figure 5E**) revealed a reduction in RT in both conditions during the experimental session [*F*(3,54) = 5.38, *p* = 0.003, $\eta_{p}^{2}$ = 0.230]. But no significant difference was found between REST and EX (95% *CI* = -0.025 to 0.002, *p* = 0.135). There was no main effect for condition [*F*(1,18) = 0.185, *p* = 0.672, $\eta_{p}^{2}$ = 0.010] or condition × time interaction [*F*(3,54) = 0.012, *p* = 0.886, $\eta_{p}^{2}$ = 0.012].

The accuracy data for Set-Shifting Task are shown in **Figure 5F**. There was no main effect for condition [*F*(1,18) = 0.278, *p* = 0.605, $\eta_{p}^{2}$ = 0.015], time [*F*(3,54) = 1.956, *p* = 0.132, $\eta_{p}^{2}$ = 0.098] or condition × time interaction [*F*(3,54) = 0.145, *p* = 0.932, $\eta_{p}^{2}$ = 0.008].

### Mean Skin Temperature, Thermal Sensation and Cognitive Performance

With regard to the correlations between MST, TS and cognitive performance, a significant positive correlation was observed between MST and TS (*p* < 0.001, *r* = 0.672). MST was also found to be correlated directly with Identification RT (*p* < 0.001, *r* = -0.341) and Two Back RT (*p* < 0.001, *r* = -0.282) and inversely with Two Back accuracy (*p* = 0.002, *r* = 0.249). There’s a significant negative correlation between TS and Identification RT (*p* = 0.001, *r* = -0.262) and Two Back RT (*p* = 0.005, *r* = -0.229). A significant positive correlation was observed between TS and Two Back accuracy (*p* = 0.001, *r* = 0.255) (**Table 2**).

**Table 2:** Correlation analysis of MST, TS and cognitive performance.

	Identification		Two back		Set-shifting		MST	TS
	RT	Accuracy	RT	Accuracy	RT	Accuracy		
**MST**								
Spearman’s rho	-0.341^∗∗∗^	0.073	-0.282^∗∗∗^	0.249^∗∗^	-0.008	0.053	1.000	0.672^∗∗∗^
Sig. (2-tailed)	<0.001	0.372	<0.001	0.002	0.927	0.514	0.000	<0.001
**TS**								
Spearman’s rho	-0.262^∗∗^	0.037	-0.229^∗∗^	0.255^∗∗∗^	-0.025	0.119	0.672^∗∗∗^	1.000
Sig. (2-tailed)	0.001	0.650	0.005	0.001	0.761	0.144	<0.001	0.000

*^∗∗^Correlation is significant at the 0.01 level.*

*^∗∗∗^Correlation is significant at the 0.001 level.*

## Discussion

The aim of the current study was to examine the immediate and sustained effects of a single bout of acute moderate exercise on multiple components of cognition in temperate and cold environmental conditions. The correlations between MST, TS and cognitive performance were also investigated. The results revealed three main findings: (1) acute moderate exercise facilitated the reaction speed of information processing and working memory in temperate environment. The facilitating effect on processing speed disappeared within 30 min, while working memory performance remained heightened 30 min after the cessation of exercise. A short-term moderate exercise intervention had no effects on cognitive flexibility; (2) in cold environment, there was also a beneficial effect on processing speed and working memory, but not on cognitive flexibility. However, the improvements in processing speed and working memory didn’t last 30 min in the recovery period; (3) there were significant correlations between MST, TS, processing speed and working memory.

### Effects of Acute Exercise on Cognition in Temperate Environments

Our findings are in line with previous studies on the transient positive effects of acute exercise on processing speed which investigated young adult subjects using moderate to high intensity ergometer cycling protocol (Kashihara and Nakahara, 2005; Audiffren et al., 2008). RT was significant shortened immediately after exercise, whereas no effect was detected 30 min after the exercise cessation. Several studies have used electromyographic (EMG) techniques to identify the mechanism underlying the effects of exercise on processing speed (Davranche et al., 2005, 2006). In their studies, adult participants cycled on an ergometer at either 50% VO_2max_ or until exhaustion. RT was fractionated into premotor time (i.e., the time interval between the onset of stimuli and the onset of EMG activity) and motor time (the time interval between the onset of EMG activity and the onset of motor response). They found that a single bout of exercise shortened motor time rather than premotor time. However, a recent study which investigated young adults employed an event-related potential (ERP) analysis revealed that exercise modulated information processing speed in multiple neural stages, and the effects of low-intensity exercise were independent of the motor stage (Bullock et al., 2015). Based on the previous literature, it can be concluded that exercise facilitates both the premotor stage and the motor stage in information processing, with a larger effect on the motor stage when the exercise intensity is higher. Exercise shortens the motor time by increasing motor unit recruitment, resulting in faster motor execution (Davranche et al., 2005, 2006). The effects disappear within a short period, which may be a plausible explanation of the transient effects in our study.

Working memory was enhanced after acute exercise. Our results are in accordance with Pontifex et al. (2009) and Quelhas Martins et al. (2013) that examined young adults and employed moderate exercise intensity protocol, but are inconsistent with Tomporowski et al. (2005) and Coles and Tomporowski (2008) which indicated either negative effects or no effects in similar population. The inconsistency may be caused by different cognitive test paradigms used in these studies, since Tomporowski et al. (2005) and Coles and Tomporowski (2008) used a Brown–Peterson Task which requires participants to resist interferences, while our study employed a n-back paradigm that mainly involves the updating component of working memory. Previous studies using n-back paradigms have also presented significant improvements after exercise in preadolescent children and young adults (Chen et al., 2016; Etnier et al., 2016). The facilitating effects on working memory remained at least 30 min after the cessation of exercise, which is in line with results from Pontifex et al. (2009). Several mechanisms have been hypothesized to explain the effects of exercise on working memory, including: increases in cerebral blood flow and cerebral oxygen consumption (Chapman et al., 2016), increase in brain derived neurotrophic factor (BDNF) (Piepmeier and Etnier, 2015) and increased concentration of central catecholamines (e.g., dopamine and norepinephrine) (McMorris and Hale, 2014). A study on the time course of BDNF changes after exercise showed improvement in circulating level for up to 60 min after the exercise cessation (Knaepen et al., 2010). This may possibly explain the 30-min sustained effects in our study.

Although reaction speed and accuracy in Set-Shifting Task showed a general increase throughout the experimental sessions, the present study failed to detect a facilitating effect of acute exercise on cognitive flexibility, which is in line with two previous studies in which young adult participants exercised at moderate intensity (Coles and Tomporowski, 2008; Wang et al., 2015) but is inconsistent with studies employing high intensity exercise protocol that indicated improvements (Barenberg et al., 2015; Berse et al., 2015; Tsai et al., 2016). Results from the current study may contribute to the previous hypothesis that exercise intensity is the potential mediators. Though a significant main effect for time was observed in the Set-Shifting Task, it seems that moderate exercise intensity as well as participants with moderate fitness level cannot evoke executive benefits. It should be noted that the Set-Shifting Task was performed following Identification and Two Back, which was 7 min after exercise. Yet the non-significant difference was not likely to be caused by the 7-min delay. Since cognitive flexibility is a higher-level cognitive function, if there was a significant decrease in RT, the facilitating effect should have last longer. However, to avoid potential errors, further research needs to have more precise time controls. In addition, the general increase in reaction speed and accuracy in our study can be explained by the distributed-learning model proposed by Pellegrini and Bjorklund (1997), which predicts that any break in a cognitively demanding task may contribute to an improvement in cognitive performance.

### Effects of Acute Exercise on Cognition in Cold Environments

Results from the current study demonstrate negative effects of cold stress on cognitive functions. Participants’ processing speed and working memory were impaired during REST in COLD, compared to the baseline level. Our findings are in consistent with Mahoney et al. (2007) and Muller et al. (2012) which reported negative effects of cold stress on CRT and memory tasks. Cold-induced decrements in cognitive performance have been hypothesized by the distraction theory, which suggests that thermal discomfort caused by cold exposure provides alternative stimuli, leads to a shift of attention from the cognitive task and results in cognitive deficits (Teichner, 1958; Enander, 1987; Muller et al., 2012). Previous studies also proposed that cognitive impairment in cold environments was associated with depletion of central catecholamines (Rauch and Lieberman, 1990; Mahoney et al., 2007; Taylor et al., 2015). Our study failed to find significant difference in Set-Shifting task between COLD and TEMP. Based on the evidence of dopaminergic contribution to cognitive flexibility function (Colzato et al., 2010; Berse et al., 2015; Steenbergen et al., 2015), we propose that changes in dopamine levels induced by neither moderate exercise intervention nor mild cold stress wouldn’t significantly influence cognitive flexibility.

To our knowledge, this is the first empirical study that investigated the time-dependent effects of acute exercise on processing speed and working memory in cold environments. Similar to TEMP, reaction speed of information processing and working memory was improved immediately after exercise in COLD. The results are in accordance with Kruk et al. (2001) in which 9 healthy soccer players cycled on an ergometer until exhaustion in 4°C and Muller et al. (2011) in which 14 young male participants completed an ergometer cycling protocol at 50% VO_2max_ in 4°C. Improvements in processing speed were blunted during the 30-min recovery period. In addition to the increase in cerebral blood flow, BDNF and central catecholamines, the exercise-induced improvements in cognitive performance in COLD are also likely to be related to the elevation of MST. Previous studies have indicated that skin temperature is the controller of TS and thermal comfort (Schlader et al., 2011a,b). Exercise-induced increase in MST may mitigate the thermal discomfort, which leads to more attention focused on the cognitive tasks and improvements in cognitive performance. In contrast to TEMP, the effect of exercise on working memory didn’t last 30 min after the cessation of exercise in COLD. Since cold exposure alters the concentration of central catecholamines, it can be deduced that the increase on the central catecholamines after exercise is blunted by cold stress during the recovery period.

### Mean Skin Temperature, Thermal Sensation and Cognitive Functions

The present study demonstrated significant correlations between MST, TS and processing speed and working memory performance. Cognitive performance was improved with elevations in MST and TS after exercise, and was impaired during REST and POST-EX with decreased MST and TS in COLD. Our findings are in line with Palinkas et al. (2005) and Mäkinen et al. (2006), and are partly in consistent with results from Watkins et al. (2014) which indicated significant correlations between MST and cognitive performance in cold (-5°C), temperate (18°C) and hot (30°C) conditions, but failed to find any correlation between TS and cognitive performance. The disassociation in their study is likely to be caused by the experimental protocol, since they administrated a 15-min break period in a thermoneutral environment halfway in the experimental session, which may result in different changes in MST and TS. Based on the previous literature and our findings, we propose that MST and TS are two independent predictors of cognitive performance when exercising in temperate and cold environments. In addition, according to the inverted-U model describing the relation of ambient temperature and performance (Hancock et al., 2007), cognitive performance will decline when the ambient temperature increases above the thermal comfort zone. When exposure to a challenging heat stress (>30°C), it is more likely that cognitive performance will follow an inverted-U shape as MST increases. Therefore, the associations between MST, TS and cognitive functions need to be reconsidered when hot environment is included.

### Limitations

There are several potential limitations that should be mentioned to this study. First, neurobiological measures were not included in our study, thus the mechanism underlying the effects of acute exercise intervention on cognitive performance could only be hypothesized and not examined directly. Further research is required to identify the mechanisms of exercise-induced time-dependent effects on cognitive performance. Second, we only examined healthy male university students with moderate fitness level. Since age, sex and fitness level may be the potential mediators of the effects of exercise on cognitive performance, further research is required to examine the generalization in other populations. Next, exercise intensity for each participant was controlled by %HRR. Individual’s HR_max_ for calculating the target HR was estimated using the equation ‘220–age’ instead of accurately measured. This may cause possible errors. Finally, the present study only administrated two time points (immediately after and 30 min after) to investigate the time course after exercise. Though results demonstrate different time-dependent effects on working memory in TEMP and COLD, more precise time courses of each cognitive function in both conditions are unknown, which need further investigation.

## Conclusion

The current results demonstrate that a single bout of acute moderate exercise has positive effects on processing speed and working memory in both temperate and cold environments, but no effects on cognitive flexibility. The time course of improvements in processing speed after exercise is less than 30 min, while effects on working memory remain heightened for at least 30 min in temperate environments. However, the effects in cold environments disappeared within 30 min. Our findings suggest that acute exercise has different time-dependent effects on university students’ cognition in temperate and conditions. Mean skin temperature and thermal sensation are associated with cognitive performance when exposure to different thermal environments. Improvements in working memory performance benefited from exercise cannot maintain for a desirable period in cold environments. It is necessary for universities to provide students with the opportunity to exercise and study in a pleasant thermal environment (e.g., central-heating), which may guarantee student’s cognitive performance and learning efficiency.

## Author Contributions

L-YJ, X-LL, YL, LC, and LG contributed to the conception and design of the study. L-YJ, YL, H-FW, and X-WS performed the experiment. L-YJ analyzed the data and drafted the manuscript. X-LL, YL, LC, and LG helped revise the manuscript and did the final approval.

## Conflict of Interest Statement

The authors declare that the research was conducted in the absence of any commercial or financial relationships that could be construed as a potential conflict of interest.

## Footnotes

1 https://cogstate.com/
